# lncRNA prostate cancer-associated transcript 18 upregulates activating transcription factor 7 to prevent metastasis of triple-negative breast cancer via sponging miR-103a-3p

**DOI:** 10.1080/21655979.2021.2003928

**Published:** 2021-12-14

**Authors:** Jinfeng Zhang, Donghua Liu, Guoming Deng, Qiuming Wang, Liang Li, Jinxiang Zhang, Heming Wu

**Affiliations:** a Department of Medical Oncology, Guangdong Provincial Key Laboratory of Precision Medicine and Clinical Translational Research of Hakka Population, Meizhou People’s Hospital (Huangtang Hospital), Meizhou Academy of Medical Sciences, Meizhou, P. R. China; b The Fifth Hospital of Xiamen, Xiamen, P.R. China; c Center for Precision Medicine, Guangdong Provincial Key Laboratory of Precision Medicine and Clinical Translational Research of Hakka Population, Meizhou People’s Hospital (Huangtang Hospital), Meizhou Academy of Medical Sciences, Meizhou, P. R. China

**Keywords:** Triple-negative breast cancer, metastasis, LncRNA PCAT18, activating transcription factor 7, MiR-103a-3p

## Abstract

Long non-coding RNA (lncRNA) prostate cancer-associated transcript 18 (PCAT18) is a potential diagnostic target for adenocarcinoma. However, its role in triple-negative breast cancer (TNBC) remains largely unknown. Based on data from an online database, a significant decline in lncRNA PCAT18 was observed in patients with TNBC subtype compared to a population with normal breast tissue. Patients with TNBC with high PCAT18 levels presented good outcomes. Patients with TNBC with high PCAT18 had a lower rate of lymph node-positive metastasis than those with low PCAT18. PCAT18-upregulation inhibited, while PCAT18-downregulation promoted, migration and expression of matrix metalloproteinases 9/2 (MMP9/MMP2) and uridylyl phosphate adenosine (uPA) in TNBC cells. Activating transcription factor 7 (ATF7) was positively associated with PCAT18, and ATF7-inhibition abrogated the anti-migration effects of PCAT18 on TNBC cells. Mechanistically, miR-103a-3p directly targeted and inhibited ATF7 expression. PCAT18 competitively sponges miR-103a-3p, promoting the expression of ATF7. Exogenous PCAT18 was associated with lower incidence of lung metastasis followed by the upregulation of ATF7, which was prevented by the treatment of miR-103a-3p mimics. Collectively, PCAT18 was expressed at low levels in TNBC, and PCAT18 could sponge miR-103a-3p and promote ATF7 expression, resulting in prevention of TNBC metastasis. Thus, PCAT18 can serve as a predictive factor for patients with metastatic TNBC.

## Graphical Abstract

**Figure:**
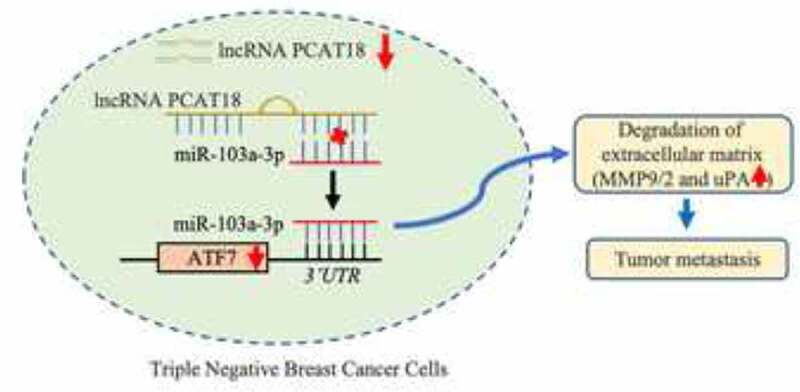

## Introduction

Breast cancer (BC) is the most common type of cancer that develops in the breast tissue. Global tumor epidemiology statistics show that, in 2020, there were an estimated 2.3 million female breast cancer cases, representing 11.7% of all cancer cases [1]. Gene expression profiling is useful to understand breast cancer biology. During the last 20 years, researchers have characterized five intrinsic molecular subtypes of breast cancer: luminal A, luminal B, HER-2 enriched, basal-like: TNBC, and claudin-low [2]. Basal-like tumors account for 15–20% of all pathological types of breast cancer [3]. It is worth noting that TNBC is typically a more aggressive type of cancer and has a worse prognosis than other subtypes of breast cancer. It also has a higher rate of distal recurrence [4]. Less than 30% of patients with metastatic TNBC survive for more than 5 years after diagnosis [5]. Clinically, broad-spectrum chemotherapy drugs, such as paclitaxel, platinum, and doxorubicin, are currently the main treatment methods for TNBC [6]. However, patients with metastatic TNBC always present resistance to chemotherapy drugs, resulting in an average survival period of only 8–13 months [7,8]. Therefore, it is of great significance to identify specific molecular targets of metastatic TNBC and to elucidate the molecular response mechanism for regulating TNBC cell migration.

Long non-coding RNAs (lncRNAs) play an important role in tumorigenesis and tumor metastasis in TNBC. lncRNAs regulate gene expression through various processes, such as chromatin modification and post-transcriptional regulation, and have become important tumor biomarkers involved in tumor pathogenesis, metastasis progression, and drug resistance in TNBC [9]. Studies have shown that the lncRNA antisense transcript NAMPT (NAMPT-AS) is significantly upregulated in TNBC tumors and is significantly positively correlated with prognosis, lymph node metastasis, distal migration, and pathological grade of patients with TNBC, suggesting that lncRNA NAMPT-AS is involved in the occurrence and distal migration of TNBC [10]. High expression of lncRNA HOTAIR can serve as a critical indicator for predicting lymph node metastasis in patients with luminal androgen receptor (LAR) subtype TNBC [11]. In addition, there are some lncRNAs with tumor-suppressive functions in TNBC [12]. In conclusion, lncRNAs have multiple functions in TNBC tumorigenesis and metastasis. The expression of lncRNA PCAT18 is negatively correlated with the size of the gastric tumor, and the upregulation of lncRNA PCAT18 can significantly inhibit the proliferation and tumor growth of gastric cancer cells [13]. It has been reported that lncRNA PCAT18 can be used as a potential biomarker for predicting the treatment effects of neuroendocrine prostate cancer [14]. Therefore, lncRNA PCAT18 is also an important target for evaluating the occurrence and therapeutic effect of adenocarcinoma, such as gastric adenocarcinoma and prostate cancer; however, its role and function in TNBC has not yet been reported.

We speculated that lncRNA PCAT18 may be involved in the migration process of TNBC. The present study aimed to clarify the effect of lncRNA PCAT18 on TNBC metastasis and illustrate the molecular mechanism by which PCAT18 regulates TNBC cell migration. These results will help us better understand the occurrence and development of metastatic TNBC.

## Materials and methods

### Data collection and analysis

The mRNA expression levels of lncRNA PCAT18 in different molecular subtypes of human breast carcinoma (BRCA) and normal tissues were analyzed using Breast Cancer Gene-Expression Miner v4.4, The Encyclopedia of RNA Interactomes (ENCORI) Starbase [15]. The probability of overall survival of breast cancer patients was determined using the Breast Cancer Gene-Expression Miner v4.4 and the online database of Kaplan-Meier Plotter [16]. The association between PCAT18, miR-103a-3p, and ATF7 expression was assessed using the ENCORI Starbase.

### Tissue collection and grouping

Samples were collected from 30 women with TNBC who visited the Meizhou People’s Hospital. Signed informed consent form was obtained from all participants, and this study was approved by the ethics committee of Meizhou People’s Hospital. Patient characteristics are shown in Table 1. None of the patients received preoperative chemotherapy or radiotherapy. Among these patients, 20 had lymph node-positive metastasis and 10 had non-lymph node metastasis. Total RNA was extracted from the patients for subsequent experiments. Based on PCAT18 expression, 21 patients were grouped into the PCAT18 high-expression group and 9 were in the PCAT18 low-expression group.

**Table 1.** Correlation between PCAT18 expression and clinicopathological characteristics of TNBC patients

	Relative PCAT18 expression		
Characteristics	Low (n = 21)	High (n = 9)	*P value*
Gender			-
Male	0	0	
Female	21	9	
Age			0.7450
≤50	8	4	
>50	13	5	
Tumor grade			0.9522
G1	3	1	
G2	6	3	
G3	12	5	
Lymph node metastasis			0.0112*
No	4	6	
Yes	17	3	
Tumor diameter (cm)			0.9250
≤5	16	7	
>5	5	2	
Pathological Type			
Noninvasive	12	4	0.5229
Invasive	9	5	

*P < 0.05 was considered statistically significant.

### Cell cultures and transfection

For the *in vitro* cell experiment, TNBC cell lines MDA-MB-231, BT549, MDA-MB 468, Hs578t, and normal mammary epithelial cell line MCF-10A were purchased from the Cell Line Bank of the Chinese Academy of Sciences (Shanghai, China). MCF-10A cells were incubated in Dulbecco’s modified Eagle’s medium (DMEM)/F12 (1:1), supplemented with 5% horse serum (16050–122; Invitrogen, USA), 100 ng/ml cholera toxin (C-8052; Sigma, USA), 10 mg/ml insulin (I-1882; Sigma, USA), 20 ng/ml epidermal growth factor (EGF; SRP3027; Sigma, USA), and 0.5 mg/ml hydrocortisone (H-0888; Sigma, USA). MDA-MB-231 and BT549 cells were cultured in Roswell Park Memorial Institute (RPMI) 1640 (SH30809.01; Hy Clone, USA) medium, and MDA-MB 468 and Hs578t cells were cultured in DMEM (Thermo Fisher Scientific, Waltham, MA, USA). Media were supplemented with 10% fetal bovine serum (FBS; Gibco; Thermo Fisher Scientific, Inc.) or 10% horse serum and 1% antibiotic–antimycotic solution at 37°C in a humidified 5% CO2 incubator. For cell transfection, the vector plasmids pcDNA3.1 and pcDNA3.1-PCAT18 were purchased from GenePharma Corporation, China. The siRNAs targeting PCAT18 (siB160229100822-1-5) and negative control siRNA (siN0000001-1-5), miR-103a-3p mimics (cat. no. miR10000101-1-5), and miR-control (miR-con; cat. no. miR1N0000001-1-5), an miR-103a-3p inhibitor (cat. no. miR20000101-1-5), and inhibitor con (cat. no. miR2N0000001-1-5) were purchased from RiboBio Corporation (Guangzhou, China). The ATF7 knockdown was performed using shRNA (5ʹ-GCTAGATTTGATGACATATTA-3ʹ, which is a sequence in the 3UTR, and 5ʹ-GTCACATTACTACGCAATG-3ʹ, which is a sequence in the CDS generated by Guangzhou RiboBio Co., Ltd. On reaching 80% confluence, the plasmid (0.8 µg), siRNA, shRNA, inhibitor, or mimics (50 nM) were transfected into the cells using Lipofectamine 2000 (Invitrogen, Thermo Fisher Scientific, Inc., USA). After transfection for 48 h at 37°C, the cells were harvested for further analysis.

### Quantitative-reverse transcription polymerase chain reaction (qRT-PCR) analysis

Total RNA from tissues and cells was extracted using TRIzol reagent (Invitrogen; Thermo Fisher Scientific, Inc.) based on the manufacturer’s instructions, and the RNA concentration was measured using the NanoDrop system. Subcellular fractionation of nuclear and cytoplasmic RNA was monitored using Norgen’s cytoplasmic and nuclear RNA purification kit (Norgen BioTek, Canada). In addition, total microRNAs were extracted from tissues and cells using the miRNeasy Serum/Plasma Kit (QIAGEN, Germany) based on the manufacturer’s instructions. Complementary DNA (cDNA) was synthesized using RevertAid first strand cDNA (Fermentas; Thermo Fisher Scientific, Inc.) or a Taqman microRNA reverse transcription kit (Applied Biosystems; Thermo Fisher Scientific, Inc.), respectively. Subsequently, qRT-PCR assays were performed using 10 µL SYBR® Green PCR Master Mix (4312704, ABI, USA) or a TaqMan microRNA assay (Applied Biosystems; Thermo Fisher Scientific, Inc.) on a Applied Biosystems 7500 Real-Time PCR System (Thermo Fisher Scientific). The primers used are presented in Table 2. U6 was used to normalize the expression levels of miR-103a-3p, while GAPDH was used to normalize the expression levels of *ATF7* and *PCAT18*. The relative levels were quantified using the 2^−ΔΔCt^ method [17].

**Table 2.** Primer sequences for qRT-PCR

Name	Primer	Sequence
GAPDH	Forward	5‘- TCAAGAAGGTGGTGAAGCAGG −3’
	Reverse	5‘- TCAAAGGTGGAGGAGTGGGT −3’
ATF7	Forward	5ʹ-CTGGAGAACTTGACGTGGCA-3’
	Reverse	5ʹ-ATCCGACATTGTTCCGGCAT-3’
PCAT18	Forward	5ʹ-AGGAGACAGGCCCCAGATTT-3ʹ
	Reverse	5ʹ-TGAAGTGCTGGGACAACGTA-3ʹ
U6	Forward	5ʹ-CTCGCTTCGGCAGCACA-3’
	Reverse	5ʹ-AACGCTTCACGAATTTGCGT-3’
MMP9	Forward	5′‐CCTCTGGAGGTTCGACGTGA‐3′
	Reverse	5′‐TAGGCTTTCTCTCGGTACTGGAA‐3′
MMP2	Forward	5′-ACTGTTGGTGGGAACTCAGAAG-3′
	Reverse	5′-CAAGGTCAATGTCAGGAGAGG-3′
MMP1	Forward	5′-ACCTGGCGCTAAAC-3′
	Reverse	5′-TGCGGGTACTCCCAC-3′
miR-103a-3p	Forward	5ʹ-ACACTCCAGCTGGGAGCAGCATTGTACAGGG-3’
	Reverse	5ʹ-TGGTGTCGTGGAGTCG-3’

### Western blot

Total protein was isolated from tissues or cells using the RIPA kit. The BCA kit was used for the protein samples for protein quantitation based on the manufacturer’s protocol. Equal proteins (20 µg) were separated by sodium dodecyl sulfate (SDS)-polyacrylamide gel electrophoresis (PAGE), followed by transfer to an activated polyvinylidene fluoride (PVDF) membrane. The activated membrane was blocked with 5% skim milk for 1 h. The membranes were incubated with primary antibodies against matrix metalloproteinases MMP9 (GeneTex, GTX31891, US), MMP2 (GeneTex, GTX55708, US), MMP1 (Abcam, ab134184, UK), uridylyl phosphate adenosine (uPA, Santa Cruz, sc-59727, US), ATF7 (Abcam, ab231786, UK), and β-actin (Sigma, A2228, USA) at 4°C overnight. Subsequently, the blots were incubated with an HRP-conjugated secondary antibody for 1 h at room temperature. The blots were visualized using the ECL chemiluminescence reagent kit (Millipore, Billerica, MA, USA) and photographed on a ChemiDoc™ MP Imaging System (Bio-Rad).

### Colony-forming assay

Briefly, a 6-well plate was covered with a mixture of 1.2% agarose and DMEM. After solidification of the DMEM, the control vector, PCAT18-overexpressing plasmid, control negative siRNA, and PCAT18-siRNA-transfected MDA-MB-231 or BT549 cells (200 cells/well) were seeded into 6-well plates and cultured in DMEM medium containing 10% FBS. After 2 weeks of incubation, the cell colonies were fixed with 4% paraformaldehyde and stained with 0.1% crystal violet at room temperature for 15 min. Images were obtained and counted using ImageJ software.

### Transwell assay

Cell migration viability was assessed using the Transwell method (PIEP12R48, Millipore, Germany). Briefly, the upper chamber was plated with TNBC cells transfected with PCAT18 overexpression plasmid, PACT18 siRNA, or ATF7 shRNA in serum-free medium, and the bottom chamber was filled with 600 μl medium containing 10% FBS. After 48 h of culture, the upper surface of each membrane was cleaned with a cotton swab, and cells that had penetrated to the bottom side of the membrane were stained with 0.1% crystal violet dye (Sigma) for 20 min. Then, the cell count was determined using a microscope (Olympus, IX51).

### Lung metastasis of breast cancer

Six-week-old female athymic nude mice (BALB/c-nu, 18–20 g) were purchased from Vital River company (Beijing, China) and housed in a specific pathogen-free isolation facility with a 12/12 h light/dark cycle. All the mice were fed ad libitum and had access to water. All experiments complied with the guidelines of the Animal Ethics Research Board and were approved by the Ethical Committee of Meizhou People’s Hospital. After 1 week, control and PCAT18-overexpressed MDA-MB-231 cells (1 × 10^6^) were resuspended in 100 μl of PBS and injected into the tail vein to establish a lung metastasis model of breast cancer. Mice injected with PCAT18-overexpressed TNBC cells were injected with a negative control mimic or miR-103a-3p mimic (50 nmol, RiBoBio, China) every week. Based on these processes, mice were divided into four groups: mice injected with blank control cells, mice injected with PCAT18-ovexpressed cells, negative control mimic-challenged mice injected with PCAT18-ovexpressed cells, and miR-103a-3p mimics-challenged mice injected with PCAT18-ovexpressed cells. All mice were euthanized 4 weeks after injection of the cancer cells. Lung tissues were harvested for subsequent experiments.

### RNA pulldown

RNA pulldown assays were performed as previously described [18]. In brief, 1 × 10^7^ MDA-MB-231 cells were collected, lysed, and treated with ultrasonic waves. A biotin-labeled miR-103a-3p probe (Sangon Biotech, Shanghai, China) or the negative control probe (Sangon Biotech, Shanghai, China) was incubated with magnetic beads (ThermoFisher, 88817) at 25℃ for 2 h to generate probe-coated beads. Subsequently, cell lysates were incubated with miR-103a-3p or oligo probes at 4℃ overnight. After washing with washing buffer, the RNA complex bound to the beads was eluted and extracted with an RNeasy Mini Kit (QIAGEN, 74104) for real-time PCR using PCAT18 and ATF7 primers. The total cell lysates served as the input and were defined as a value of 1.

### Histological staining

Four weeks after tumor cell inoculation, lungs were collected and fixed in 4% paraformaldehyde for more than 24 h. Lung tissues were dehydrated with graded ethanol, immersed in xylene, and embedded in paraffin. Sections of 5-μm thickness were stained with hematoxylin and eosin (H&E; Beyotime Institute of Biotechnology, China). Representative fields for each group were photographed using an optical microscope (Olympus IX71, Olympus Corporation).

### Immunofluorescence staining

Paraffin sections (5 μm) of the tumor and lung tissue were subjected to immunofluorescence staining. Briefly, primary antibodies against α-SMA (Abcam, UK) were incubated with sections overnight at 4°C. Subsequently, Alexa Fluor 488-labeled IgG (dilution 1:200; Molecular Probes) was used as the secondary antibody. After staining with DAPI (dilution 1:300; Beyotime Institute of Biotechnology), the slices were photographed under a inverted microscope (IX51, Olympus, Japan).

### miRNA screening

Using the ENCORI Starbase, 214 candidate miRNAs targeting ATF7 were identified. Seven candidate miRNAs targeting PCAT18 were identified. The miRNAs that could directly bind to ATF7 and PCAT18 were subsequently screened using a Venn diagram.

### Dual luciferase reporter gene experiment

The wild-type (WT) and mutant sequence (MUT) (the region bound by miR-103a-3p) of PCAT18 and ATF7 were sub-cloned into the luciferase reporter vector (pGL3.1 reporter plasmid). PCAT18-WT/MUT plasmid transfected cells or ATF7-WT/MUT transfected cells were challenged with miR-103a-3p mimics or negative mimics for an additional 48 h, respectively. Subsequently, firefly and Renilla luciferase activities were measured using the dual-luciferase reporter assay system (Promega, Madison, WI, USA) and analyzed using a microplate reader. The ratio of firefly fluorescence to Renilla fluorescence intensity indicated the relative fluorescence activity.

### Statistical analysis

All assays were performed at least three times. Overall survival was estimated using the Kaplan–Meier method. Comparisons between groups were analyzed with GraphPad Prism 6.0, using a paired Student’s t-test or one-way ANOVA. Association analysis was performed using the Pearson’s correlation coefficient. All tests of significance were set at p < 0.05.

## Results

In the present study, we speculated that lncRNA PCAT18 may be involved in the migration process of TNBC. We found that low expression of lncRNA PCAT18 in TNBC and PCAT18 negatively regulated the migration ability of TNBC cells. Mechanistically, PCAT18 competitively sponges miR-103a-3p, and miR-103a-3p directly targets and inhibits ATF7 expression, resulting in the reduction of ATF7. These results will help us better understand the occurrence and development of metastatic TNBC.

## PCAT18 is expressed at a low level in TNBC

**Figure 1.**
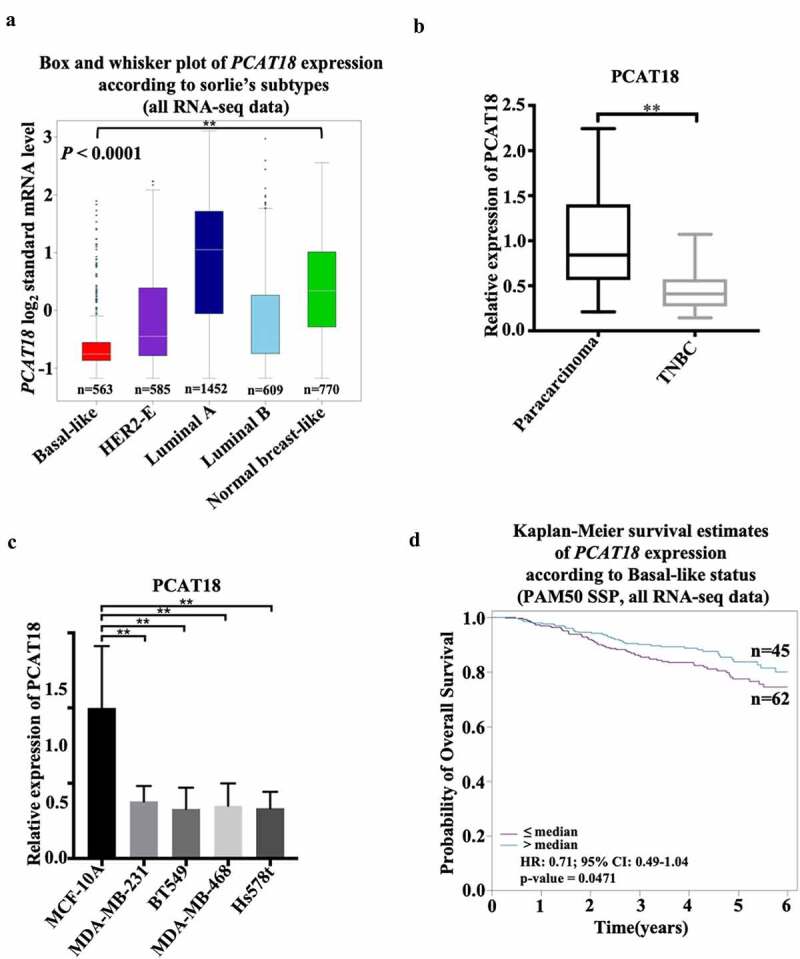
**Expression and clinical value of lncRNA PCAT18 in TNBC**. (a) Differential expressions of lncRNA PCAT18 in different subtypes of breast cancer were determined by using the Breast Cancer Gene-Expression Miner v4.4 database. Basal like, triple-negative breast cancer (TNBC); HER2-E, HER2-overexpression; ** indicates Basal-like vs. normal breast. (b) PCAT18 expression in TNBC specimens (n = 30) and paracarcinoma as determined by qRT-PCR. ** indicates TNBC vs. paracarcinoma. (c) PCAT18 expression in normal mammary epithelial cell MCF-10A, Basal-like MDA-MB-231, MDA-MB-468, Hs578t and BT549 cells as measured by qRT-PCR. ** indicates Basal-like cells vs. MCF-10A cells. (d) Patients with TNBC were divided into two groups based on PCAT18 expression status. Subsequently, the probability of overall survival (OS) as analyzed with Kaplan-Meier Plotter online database. Data are shown as the mean ± SD. Assays were performed in triplicate. *P < 0.05, **P < 0.01

To explore the role of lncRNA PCAT18 in TNBC development, the expression pattern of PCAT18 was determined using the online database of Breast Cancer Gene-Expression Miner (v4.4) and ENCORI Starbase. Based on the analysis of ENCORI Starbase, no differential expression of PCAT18 was observed between samples from normal subjects (n = 113) and those from patients with breast cancer (BRCA, n = 1104) (Fig. S1A). However, compared to normal samples (normal breast-like, n = 770), a robust decline in PCAT18 was found in TNBC samples (basal-like, n = 563) (Figure 1(a)). PCAT18 reduction was also confirmed in TNBC tissues (n = 30), in contrast to the corresponding paired paracarcinoma tissues (Figure 1(b)). In human TNBC cells, including MDA-MB-231, BT549, MDA-MB-468, and Hs578t, PCAT18 expression also declined significantly when compared to its expression in normal mammary epithelial cell line MCF-10A (Figure 1(c)). The data indicated a low level of PCAT18 expression in TNBC. In addition, the diagnostic and prognostic value of PCAT18 in TNBC was evaluated in this study. In the collected fresh TNBC specimens (n = 30), no associations were observed between PCAT18 levels and clinicopathological characteristics, including gender, age, tumor grade, tumor diameter, and pathology of patients with TNBC (Table 1). In contrast, patients with TNBC with high PCAT18 presented a good outcome compared to patients with TNBC who had low PCAT18 (Figure 1(d)), while a positive result was not observed in all subtypes of breast cancer using ENCORI Starbase (Fig. S1B). PCAT18 may be a good prognostic predictor for TNBC.

## PCAT18 affects cell migration and extracellular matrix protein expression of TNBC cells

**Figure 2.**
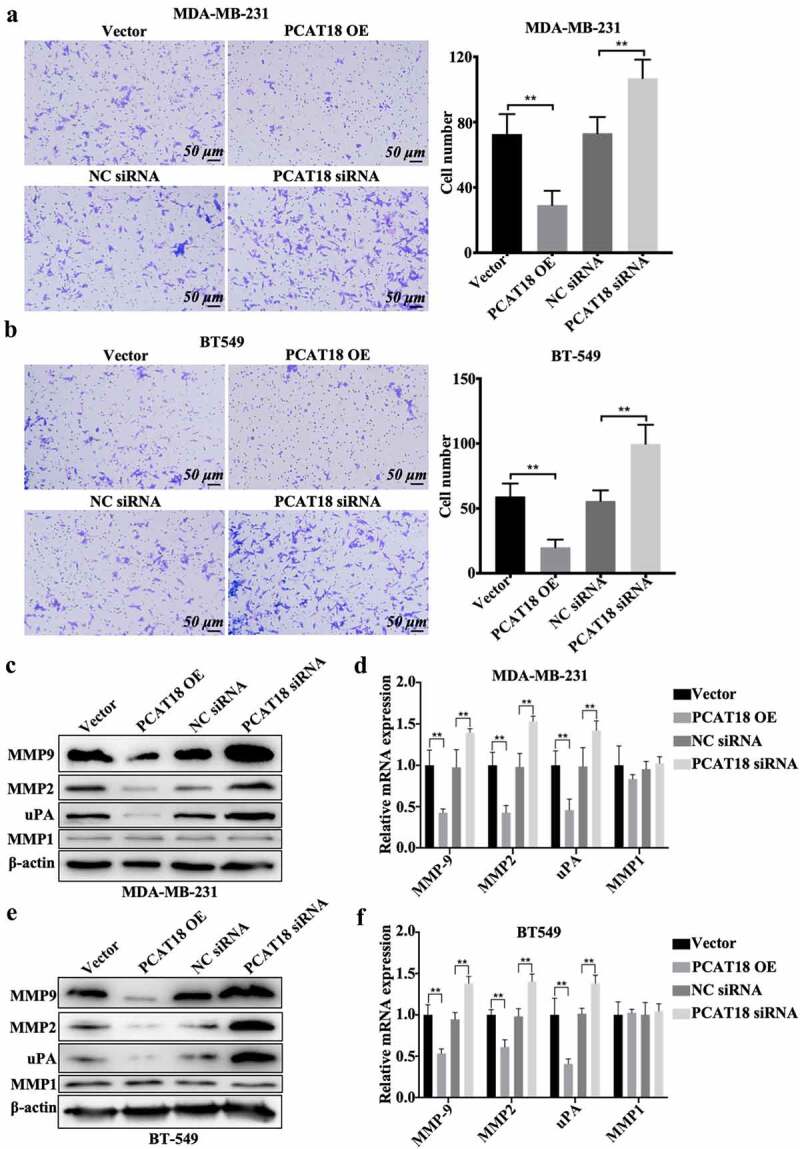
**Effects of PCAT18 on TNBC cell migration**. MDA-MB-231 and BT549 cells were transfected with PCAT18 overexpression plasmid, siRNA, and the corresponding control vector/siRNA. PCAT18-overexpressed MDA-MB-231 and BT549 cells were screened. Migration of PCAT18-overexpressed and PCAT18-depleted MDA-MB-231 cells (a) and PCAT18-overexpressed and PCAT18-depleted BT549 cells (b) as determined by Transwell assay. Relative quantification of migrated cells as shown in the right panel. ** indicates PCAT18-overexpression or PCAT18 siRNA vs. Vector or NC siRNA. NC, negative control. Scale bar: 50 μm. Protein expressions (c) and transcriptional levels (d) of MMP9, MMP2, MMP1, and uPA in PCAT18-overexpressed and PCAT18-depleted MDA-MB-231 cells as measured by WB and qRT-PCR, respectively. Protein expressions (e) and transcriptional levels (f) of MMP9, MMP2, MMP1, and uPA in PCAT18-overexpressed and PCAT18-depleted BT549 cells as measured by WB and qRT-PCR, respectively. Data are shown as the mean ± SD. Assays were performed at least three times. *P < 0.05, **P < 0.01

Although there was no association between PCAT18 expression and several clinicopathological characteristics of patients with TNBC, patients with TNBC with low expression of PCAT18 showed more lymph node-positive metastasis (n = 17) than those with high PCAT18 (n = 3) (Table 1), implying the functional role of PCAT18 in TNBC cell migration. To verify this hypothesis, PCAT18-overexpressed TNBC cell lines or PCAT18-knocked TNBC cells were generated. In MDA-MB-231 and BT549 cells, the transfection efficiency of PCAT18-overexpression plasmid and siRNA targeting PCAT18 were identified by qRT-PCR (Fig. S2A). As shown in Figure 2(a,b), PCAT18 overexpression notably suppressed cell migration and the number of TNBC cells that migrated to the bottom chamber, while PCAT18 knockdown significantly activated cell migration behavior (Figure 2(a,b)). Interestingly, protein levels and transcriptional levels of extracellular matrix-related molecules, including matrix metalloproteinases MMP9/MMP2 and uridylyl phosphate adenosine (uPA) significantly declined, accompanied by the upregulation of PCAT18, and were upregulated by the knockdown of PCAT18 in MDA-MB-231 and BT549 cells (Figure 2(c–f)). However, altered PCAT18 expression had no effect on MMP1 levels (Figure 2(c–f)), and had a slight effect on cell proliferation and colony forming efficiency of TNBC cells (Fig. S2B-S2C). Degradation of the basement membrane and extracellular matrix structures are important features of the metastatic process of breast cancer [19]. The activities of MMP9/2 and uPA are mainly responsible for the degradation of the basement membrane and extracellular matrix [20]. Therefore, PCAT18-mediated remission of TNBC cell migration may be implicated in remodeling of the extracellular matrix.

## The positive association between PCAT18 and ATF7 in TNBC

**Figure 3.**
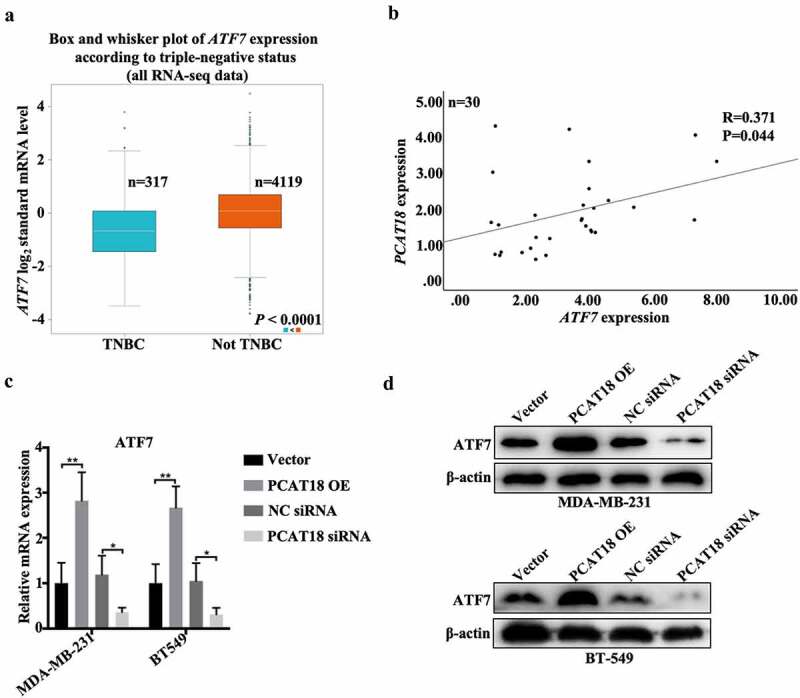
**Association between PCAT18 and ATF7 in TNBC**. (a) Differential expressions of ATF7 in TNBC (n = 317) and not TNBC (4119) specimens were determined by using the Breast Cancer Gene-Expression Miner v4.4 database. (b) The association between PCAT18 and ATF7 expression in fresh TNBC specimens as analyzed by SPSS. R, Pearson coefficient. (c) MDA-MB-231 and BT549 cells were transfected with PCAT18 overexpression plasmid, siRNA, and the corresponding control vector/siRNA. The mRNA levels of ATF7 in MDA-MB-231 and BT549 cells as determined by qRT-PCR. ** indicates PCAT18-overexpression or PCAT18 siRNA vs. Vector or NC siRNA. (d) Protein levels of ATF7 in MDA-MB-231 and BT549 cells as determined by WB. Data are shown as the mean ± SD. Assays were performed at least three times. *P < 0.05, **P < 0.01

ATF family proteins affect the migration of tumor cells by regulating the degradation of the extracellular matrix [21]. In the present study, low expression of ATF7 was found in breast cancer (n = 1104) specimens compared to normal subjects (n = 113) (Fig. S3A) and was also verified in TNBC tissues (n = 317) compared to non-TNBC tissues (n = 4119) (Figure 3(a)). Notably, ATF7 expression was positively associated with PCAT18 expression in both breast cancer tissues and TNBC-specific specimens (Fig. S3B and 3B). Additionally, transcription and translation levels of ATF7 robustly increased with the challenge of exogenous PCAT18, and dramatically decreased with the depletion of PCAT18 in both MDA-MB-231 and BT549 cells (Figure 3(c,d)). Thus, PCAT18 expression was positively associated with ATF7 expression in TNBC.

## ATF7 knockdown abolishes the effects of PCAT18 on migration in TNBC cells

**Figure 4.**
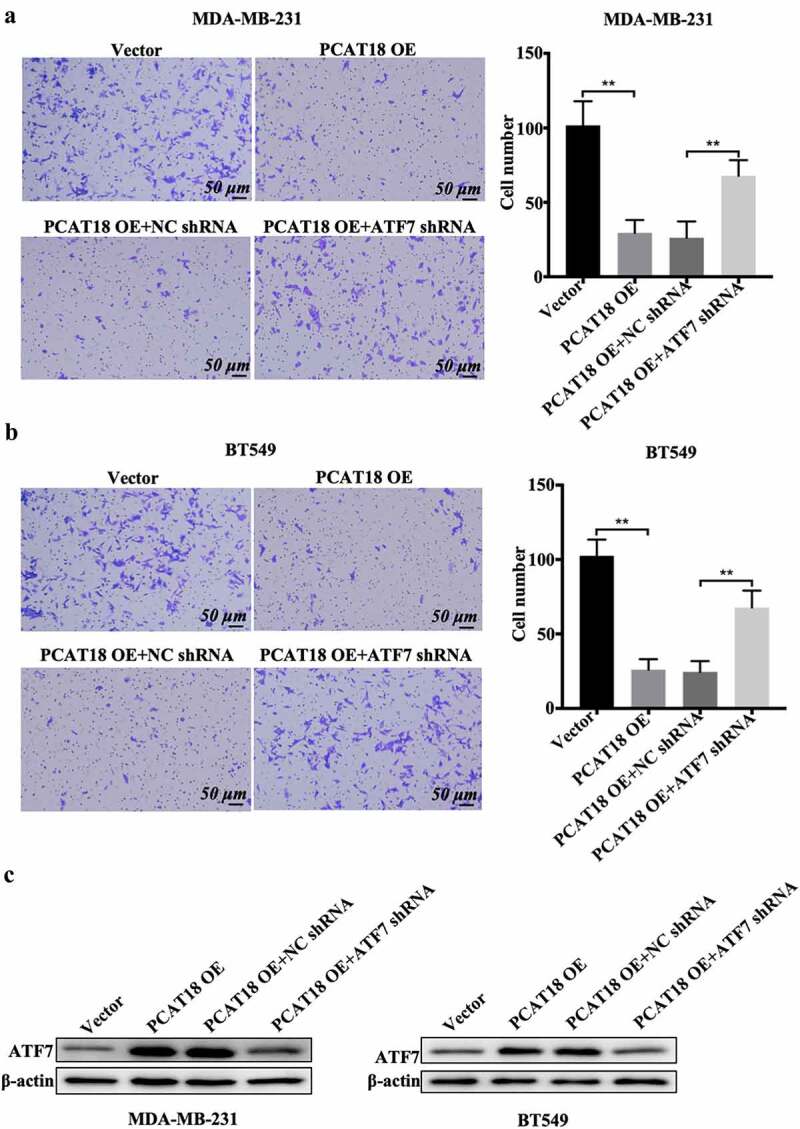
**Effects of ATF7 on PCAT18-mediated TNBC cell migration**. MDA-MB-231 and BT549 cells were transfected with vector, PCAT18-overexpression plasmid, combination of PCAT18-overexpression plasmid and NC shRNA, and combination of PCAT18-overexpression plasmid and ATF7 shRNA. PCAT18-overexpressed MDA-MB-231 and BT549 cells were screened. Subsequently, migration of MDA-MB-231 cells (a) and BT549 cells (b) as measured by Transwell assay. Relative quantification of migrated cells as shown in the right panel. Scale bar: 50 μm. (c) Protein expression of ATF was verified by WB assay in these cells. ** indicates PCAT18-overexpression or PCAT18-overexpression with ATF7 shRNA vs. Vector or PCAT18-overexpression with NC shRNA. NC, negative control. Data are shown as the mean ± SD. Assays were performed at least three times. *P < 0.05, **P < 0.01

The functional role of ATF7 in PCAT18-mediated alteration of cell migration was investigated. As expected, PCAT18 upregulation inhibited cell migration and the number of migratory MDA-MB-231 and BT549 cells (Figure 4(a,b)). The combined transfection of PCAT18 overexpression plasmid and negative control short hairpin RNA (shRNA) did not affect the role of exogenous PCAT18 on cell migration (Figure 4(a,b)). In contrast, shRNA-mediated depletion of ATF7 significantly abolished the PCAT18-upregulation-blocked migration capability of TNBC cells (Figure 4(a,b)). ATF7 expression was also verified by WB in TNBC cells (Figure 4(c)). Therefore, ATF7 is required for the PCAT18-mediated inhibition of migration. These data further prove that ATF7-induced remodeling of the extracellular matrix may be involved in the PCAT18-regulated migration process.

## The binding of PCAT18 to miR-103a-3p results in an increase in ATF7

**Figure 5.**
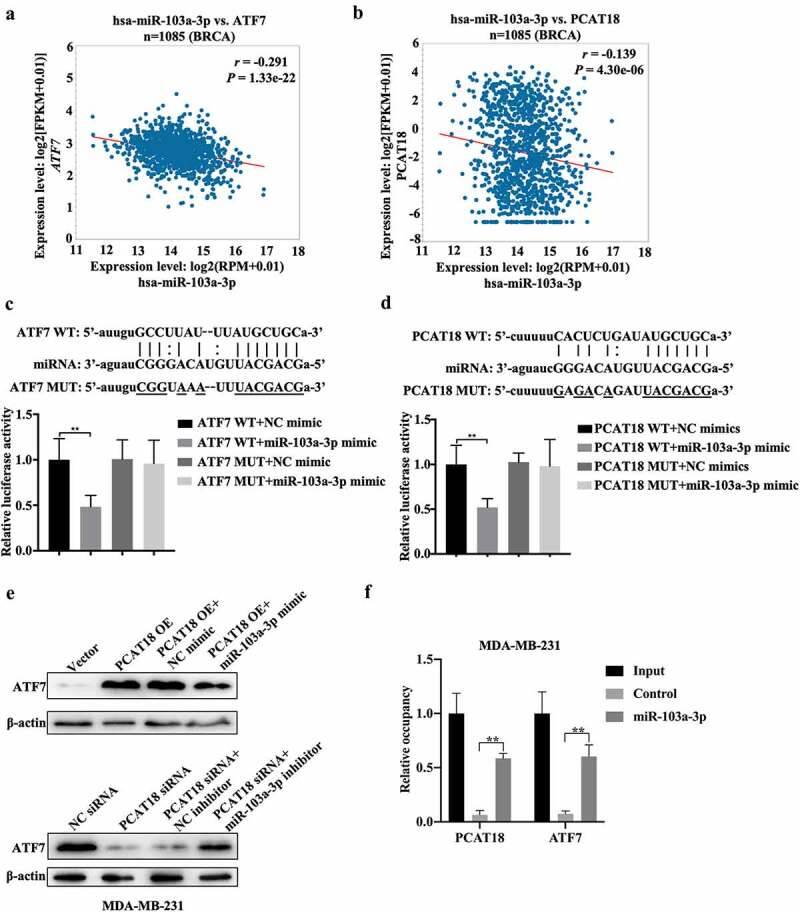
**Regulatory mechanism among PCAT18, miR-103a-3p and ATF7**. (a) The association between miR-103a-3p and ATF7 in Breast Cancer as analyzed using ENCORI Starbase. (b) The association between miR-103a-3p and PCAT18 in Breast Cancer as analyzed using ENCORI Starbase. (c) The binding sites between ATF7 and miR-103a-3p as analyzed using ENCORI Starbase. The luciferase activity was determined using the corresponding kits following transfection with ATF7 WT and ATF7 MUT reporter plasmid in the presence of NC mimic or miR-103a-3p mimic. ** indicated cells transfected with ATF7 WT and cells transfected miR-103a-3p mimic vs. ATF7 WT and NC mimic. WT, wild type; MUT, mutation. (d) The binding sites between PCAT18 and miR-103a-3p as analyzed using ENCORI Starbase. After being transfected with PCAT18 WT and PCAT18 MUT reporter plasmid in the presence of NC mimic or miR-103a-3p mimic, the luciferase activity was determined using the corresponding kits. ** indicates cells transfected with PCAT18 WT and cells transfected miR-103a-3p mimic vs. PCAT18 WT and NC mimic. WT, wild type; MUT, mutation. (e) MDA-MB-231 cells were transfected with vector, PCAT18 overexpression plasmid, combination of PCAT18-overexpression plasmid and NC mimic, and combination of PCAT18-overexpression plasmid and miR-103a-3p mimic. Consistently, MDA-MB-231 cells were transfected with NC siRNA, PCAT18 siRNA, combination of PCAT18 siRNA and NC inhibitor, and combination of PCAT18 siRNA and miR-103a-3p inhibitor. Protein levels of ATF7 in MDA-MB-231 and BT549 cells as determined by WB. (f) Biotin labeled miR-103a-3p probe or control probe were used for incubation with cell lysates. Subsequently, expression of ATF7 and PCAT18 in RNA-binding complex were verified by qRT-PCR. Data are shown as the mean ± SD. Assays were performed at least three times. *P < 0.05, **P < 0.01

The regulatory role of PCAT18 in ATF7 expression was explored in subsequent experiments. We hypothesized that PCAT18 could serve as a competing endogenous RNA by sponging miRNAs, thereby regulating the expression of ATF7. Since the model of competing endogenous RNA by sponging miRNAs is based on cytoplasmic lnRNA/circRNA, the expression of nuclear and cytoplasmic lncRNA PCAT18 was determined by qRT-PCR in MDA-MB-231 cells. The results indicated that PCAT18 was expressed in both the cytoplasm and nucleus, with higher levels in the nucleus (Fig. S4A). Subsequently, using ENCORI Starbase online database and cluster analysis, the Wayne chart presented seven miRNAs that could bind to ATF7 and PCAT18, including hsa-miR-135a-5p, hsa-miR-125b-5p, hsa-miR-135b-5p, hsa-miR-125a-5p, hsa-miR-107, hsa-miR-3194-3p, and hsa-miR-103a-3p (Fig. S4B). Combined with the association analysis using ENCORI Starbase, only hsa-miR-103a-3p appeared to be significantly negatively associated with PCAT18 and ATF7 in the breast cancer database (Figure 5(a,b)). Subsequently, a reporter gene assay was employed to verify the direct binding of miR-103a-3p to ATF7 or PCAT18. The relative luciferase activity of cells transfected with the ATF7 WT reporter plasmid was notably repressed by the miR-103a-3p mimic, while that in cells transfected with the ATF7 MUT reporter plasmid was not affected by the miR-103a-3p mimic, indicating that miR-103a-3p could negatively regulate the expression of ATF7 (Figure 5(c)). Consistently, the luciferase activity of PCAT18 WT reporter plasmid-transfected cells was inhibited by exogenous miR-103a-3p, but not PCAT18 MUT reporter plasmid-transfected cells (Figure 5(d)). Moreover, miR-103a-3p mimic-mediated the upregulation of miR-103a-3p inhibition, while miR-103a-3p inhibitor-mediated the downregulation of miR-103a-3p promoted the expression of PCAT18 in MDA-MB-231 cells (Fig. S4C-S4D), further suggesting that PCAT18 competitively binds to miR-103a-3p. Notably, PCAT18 overexpression induced by the increase of ATF7 was prevented by treatment with the miR-103a-3p mimic, while PCAT18 downregulation-mediated decline of ATF7 was recovered by transfection with the miR-103a-3p inhibitor (Figure 5(e)). Pull-down experiments further confirmed the direct binding of miR-103a-3p to PCAT18 or ATF7 (Figure 5(f)). It is possible that the competitive binding of PCAT18 to miR-103a-3p restrained the inhibitory role of miR-103a-3p on ATF7 transcription, leading to the elevation of ATF7 protein in TNBC cells.

## miR-103a-3p abrogates the effects of PCAT18 on lung metastasis of TNBC cells

**Figure 6.**
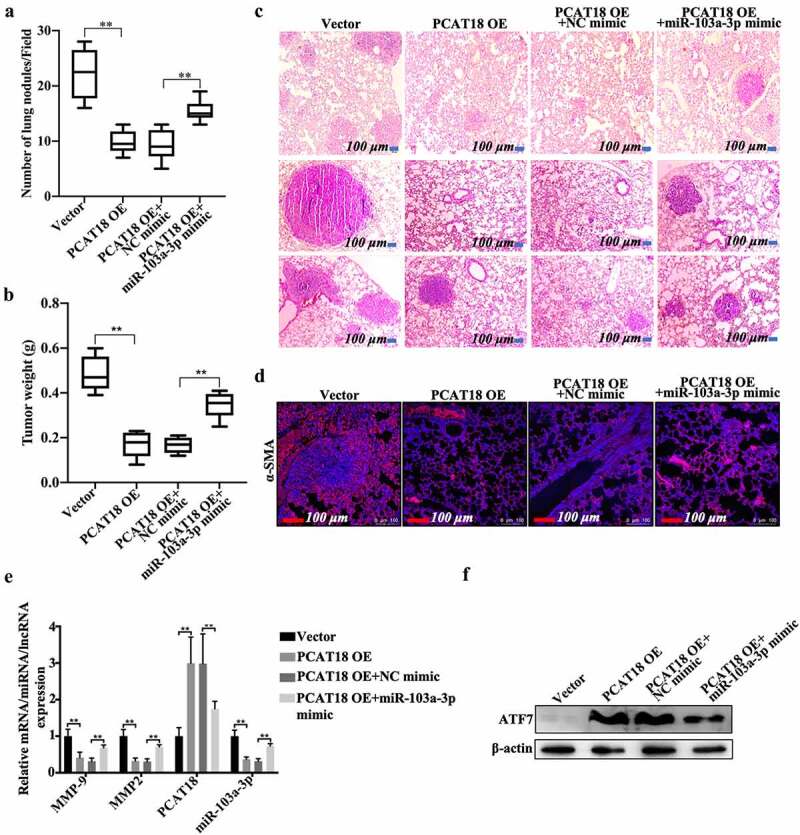
**Effects of PCAT18 and miR-103a-3p on mice model of lung metastasis of breast cancer**. Control or PCAT18-overexpressed MDA-MB-231 cells were injected into the tail vein to establish a lung metastasis model of breast cancer. During modeling, miR-103a-3p mimic was also injected into the tail vein every week. After 4 weeks, lung tissues were harvested. (a) Quantification of pulmonary metastatic nodules. (b) Tumor weights of the four groups of mice. (c) After 4 weeks, lung tissues were stained with HE solution. Dark red areas indicated the metastases in the lungs. Scale bar, 100 μm. (d) Immunofluorescent staining of α-SMA in the lung tissues. The red indicates α-SMA-positive signals. (e) In the lung tissues of the four groups of mice, mRNA of MMP9, MMP2, PCAT18, and miR-103a-3p as determined by qRT-PCR. ** indicates cells transfected with PCAT18 overexpression plasmid or the combination of PCAT18 overexpression plasmid and miR-103a-3p mimic vs. cells transfected vector or the combination of PCAT18 overexpression plasmid and NC mimic. (f) Protein levels of ATF7 in MDA-MB-231 and BT549 cells as determined by WB. Data are shown as the mean ± SD. Assays were performed at least three times. *P < 0.05, **P < 0.01

The PCAT18/miR-103a-3p/ATF7 signaling axis was also demonstrated in a lung metastasis model of breast cancer. Approximately 22 tumor nodules were detected in the vector-administered model group, while nine nodules were found in the PCAT18-overexpressed group (Figure 6(a)). Compared to the PCAT18-overexpressed group (NC mimic treatment, approximately 9 nodules), 15 nodules were observed after exposure to the miR-103a-3p mimic (Figure 6(a)). Moreover, tumor weight significantly decreased in the PCAT18-overexpressed group compared to the control group, while miR-103a-3p mimic management notably counteracted the anti-tumor growth effect produced by PCAT18 overexpression (Figure 6(b)). HE staining results showed marked enhancement of metastasis in the lung tissues of the control group (Figure 6(c), left). However, only a few metastatic lesions were observed in the lung tissue of mice injected with PCAT18-overexpressed cells (Figure 6(c), second left). In contrast, the injection of miR-103a-3p mimic abolished the role of PCAT18 in lung metastasis (Figure 6(c), the fourth image vs. the third image). Subsequent immunofluorescence staining in lung tissue indicated that the number of α-SMA-positive cells in the PCAT18-overexpressed group was significantly lower than that in the control group, while miR-103a-3p mimic challenge significantly reversed this phenomenon (Figure 6(d)). In lung tissues, the relative expression of MMP9, MMP2, and miR-103a-3p was notably reduced, accompanied by the upregulation of PCAT18 (Figure 6(e)). However, a PCAT18-mediated decrease in MMP9 and MMP2 was recovered by injection of miR-103a-3p mimic, which also resulted in the decline of PCAT18 in lung metastatic lesions (Figure 6(e)). Additionally, protein expression of ATF7 increased significantly in the PCAT18-overexpressed group and declined in miR-103a-3p in the PCAT18 OE group (PCAT18-overexpressed group) (Figure 6(f)). Collectively, PCAT18-mediated improvement in lung metastasis of TNBC cells was impaired by miR-103a-3p. It is possible that the PCAT18/miR-103a-3p/ATF7 signaling axis plays a decisive role in the metastatic process of TNBC.

## Discussion

Breast cancer accounts for 15% of all cancer-related mortality in women. Indeed, breast cancer appears to have the largest number of new cases and the highest mortality among all types of cancer [22]. Currently, the use of surgery combined with radiotherapy and chemotherapy, anti-estrogen drugs, and targeted drugs, such as trastuzumab, have made promising advancements in the clinical treatment of breast cancer. However, due to the lack of ER, PR, and HER2 on the surface of cancer cells in patients with TNBC, there is a lack of clear molecular targets for treating these patients [23]. Additionally, the high heterogeneity of TNBC tissues is another negative factor for TNBC using gene-based targeted therapy. Although patients with TNBC have been proven to have a stable and positive response to immunotherapy (such as PD-1/PD-L1 inhibitors), clinically, patients with tumors and metastatic TNBC show substantially low sensitivity to immunosuppressive therapy, with only a 5% response rate [24]. Therefore, it is important to understand the underlying regulatory mechanisms of metastatic TNBC. In the present study, PCAT18 expression declined and was negatively associated with the overall survival of patients with TNBC. Additionally, patients with TNBC with low PCAT18 levels were more likely to develop lymph node-positive metastasis, suggesting that PCAT18 may serve as a critical regulator for patients with metastatic TNBC.

lncRNA PCAT18 expression has a significant effect in tissue-specific downregulation in gastric cancer compared to normal tissues and can be used as a diagnostic factor for gastric cancer [25]. PCAT18 is upregulated in acute myeloid leukemia (AML) samples and may act as a diagnostic and prognostic biomarker for AML [26]. In this study, low expression of PCAT18 was also identified in TNBC and TNBC cells. Patients with high PCAT18 had a better prognosis than those with low PCAT18, indicating that PCAT18 may be a predictive factor for TNBC. However, PCAT18 is also abnormally expressed in HER2-E and luminal A/B subtypes of breast cancer (Figure 1(a)). Whether PCAT18 plays a functional role and can serve as a diagnostic biomarker for TNBC remains to be investigated. PCAT18 is also abnormally expressed in migratory prostate cancer and can be used as a potential therapeutic target for the treatment of metastatic prostate cancer [27]. PCAT18 can upregulate TP53INP1 expression to inhibit metastasis of gastric cancer by sponging miR-301a [28]. These data suggest that PCAT18 plays an important role in the metastatic process of adenocarcinoma. In this study, patients with TNBC with lymph node-positive metastasis presented with low expression of PCAT18. PCAT18 upregulation was inhibited, whereas PCAT18 downregulation promoted TNBC cell migration. Our results indicate the regulatory role of PCAT18 in TNBC metastasis. Alteration of PCAT18 expression causes the impairment of colorectal cancer (CRC) and gastric cancer proliferation [28,29]. However, PCAT18 had a slight role in the viability of TNBC cells, implying that PCAT18 is mainly responsible for cell migration in TNBC.

ATF7 is significantly correlated with the prognosis of colon cancer and is involved in the proliferation, apoptosis, and survival of various tumor cells [30,31]. Importantly, ATF family proteins play a key role in cancer cell migration. ATF family proteins can promote epidermal-mesenchymal transformation (EMT), invasion, and migration of tumor cells through degradation of the extracellular matrix [21,32]. Leptin can promote invasion and lung metastasis of breast cancer cells by activating the PI3K/Akt-ATF-2 signaling pathway [33]. The c-Jun/ATF-3 complex can bind to the matrix metalloproteinase (MMP-13) promoter AP-1 to regulate the invasion of breast cancer cells [34]. In this study, ATF7 was also lowly expressed in TNBC specimens compared to normal subjects and was positively associated with PCAT18 levels. PCAT18 positively regulated the expression of ATF7, and knockdown of ATF7 abolished exogenous PCAT18-mediated migration inhibition in TNBC cells. PCAT18 overexpression decreased the activities of matrix metalloproteinases, followed by a reduction in cell migration. It is possible that the negative regulation of ATF7 on the function of PCAT18 is implicated in matrix metalloproteinases.

lncRNAs or circRNAs enrich miRNAs mainly through the model of competing endogenous RNA (ceRNAs); indeed, cytoplasmic lncRNAs/circRNAs containing miRNA binding sites interact with miRNAs through their seed sequences [35]. LncRNA AFAP1-AS1 promotes the proliferation of pituitary adenoma cells by regulating miR-103a-3p and the downstream PI3K/Akt signaling pathway [36]. In glioma stem cells, lncRNA 00152 plays a biological role in inhibiting the proliferation, migration, and invasion of tumor stem cells by sponging miR-103a-3p [37]. MiR103A-3p also participates in circRNA Dicer1-mediated glioma endothelial cell migration and circRNA TCF25 regulated the invasion and migration of bladder cancer cells [38,39]. The above studies suggest that miR-103a-3p plays an important role in tumor migration through the ceRNA mechanism. Combined with the online database, miR-103a-3p was identified as a potential sponge of PCAT18. Studies have shown that miRNA-103A-3p can significantly promote the proliferation of gastric cancer cells by targeting and inhibiting the expression of ATF7 [40]. Our data showed the occurrence of binding of miR-103a-3p to ATF7 in TNBC cells. Also, there was a negative association between miR-103a-3p and ATF7 or between PCAT18 and miR-103a-3p. PCAT18 overexpression-induced increase in ATF7 expression was abrogated by miR-103a-3p mimics. These data confirmed that PCAT18 could sponge miR-1031-3p, which blocked the inhibitory role of miR-103a-3p on ATF7, resulting in the increase in ATF7. Indeed, PCAT18 upregulation mediated the improvement in lung metastasis in breast cancer, which was also aided by injection of miR-103a-3p mimic, followed by the decline in ATF7. Thus, PCAT18 regulated TNBC cell migration, possibly through the miR-103a-3p/ATF7 signaling pathway. However, other miRNAs can also be enriched by PCAT18; thus, other regulatory mechanisms of TNBC metastasis implicated in PCAT18 should be explored in future studies.

## Conclusion

In summary, lncRNA PCAT18 can be used as a potential prognostic biomarker for patients with metastatic TNBC. PCAT18 negatively affects TNBC cell migration through the miR-103a-3p/ATF7 signaling pathway. Our findings lay a theoretical foundation for the application of lncRNAs (PCAT18) in treating patients with metastatic TNBC, providing important clinical significance for improving the treatment status of TNBC.

## Supplementary Material

Supplemental Material

## Data Availability

All data generated or analyzed during this study are included in this published article and its additional files.

## References

[1] Sung H, Ferlay J, Siegel RL, et al. Global cancer statistics 2020: GLOBOCAN estimates of incidence and mortality worldwide for 36 cancers in 185 countries. CA Cancer J Clin. 2021;71(3):209–249. 33538338 10.3322/caac.21660

[2] Cancer Genome Atlas, N. Comprehensive molecular portraits of human breast tumours. Nature. 2012;490(7418):61–70. 23000897 10.1038/nature11412 PMC3465532

[3] O’Brien KM, Cole SR, Tse CK, et al. Intrinsic breast tumor subtypes, race, and long-term survival in the Carolina Breast Cancer Study. Clin Cancer Res. 2010;16(24):6100–6110. 21169259 10.1158/1078-0432.CCR-10-1533 PMC3029098

[4] Hayashi Y, Satake H, Ishigaki S, et al. Kinetic volume analysis on dynamic contrast-enhanced MRI of triple-negative breast cancer: associations with survival outcomes. Br J Radiol. 2019;93(1106):20190712. 10.1259/bjr.20190712 PMC7055451 31821036

[5] Dent R, Trudeau M, Pritchard KI, et al. Triple-negative breast cancer: clinical features and patterns of recurrence. Clin Cancer Res. 2007;13(15):4429–4434. 17671126 10.1158/1078-0432.CCR-06-3045

[6] Kumar P, Aggarwal R. An overview of triple-negative breast cancer. Arch Gynecol Obstet. 2016;293(2):247–269. 26341644 10.1007/s00404-015-3859-y

[7] Den Brok WD, Speers CH, Gondara L, et al. Survival with metastatic breast cancer based on initial presentation, de novo versus relapsed. Breast Cancer Res Treat. 2017;161(3):549–556. 28000014 10.1007/s10549-016-4080-9

[8] Kassam F, Enright K, Dent R, et al. Survival outcomes for patients with metastatic triple-negative breast cancer: implications for clinical practice and trial design. Clin Breast Cancer. 2009;9(1):29–33. 19299237 10.3816/CBC.2009.n.005

[9] Bhan A, Soleimani M, Mandal SS. Long noncoding RNA and cancer: a new paradigm. Cancer Res. 2017;77(15):3965–3981. 28701486 10.1158/0008-5472.CAN-16-2634 PMC8330958

[10] Zhang HW, Zhang N, Liu Y, et al. Epigenetic regulation of NAMPT by NAMPT-AS drives metastatic progression in Triple-Negative Breast Cancer. Cancer Res. 2019;79(13):3347–3359. 30940661 10.1158/0008-5472.CAN-18-3418

[11] Collina F, Aquino G, Brogna M, et al. LncRNA HOTAIR up-regulation is strongly related with lymph nodes metastasis and LAR subtype of Triple Negative Breast Cancer. J Cancer. 2019;10(9):2018–2024. 31205562 10.7150/jca.29670 PMC6548158

[12] Li S, Zhou J, Wang Z, et al. Long noncoding RNA GAS5 suppresses triple negative breast cancer progression through inhibition of proliferation and invasion by competitively binding miR-196a-5p. Biomed Pharmacother. 2018;104:451–457. 29793177 10.1016/j.biopha.2018.05.056

[13] Chen P, Zhao X, Wang H, et al. The down-regulation of lncRNA PCAT18 promotes the progression of gastric cancer via MiR-107/PTEN/PI3K/AKT signaling pathway. Onco Targets Ther. 2019;12:11017–11031. 31853187 10.2147/OTT.S225235 PMC6916702

[14] Crea F. Neuroendocrine prostate cancer: long noncoding RNAs to treat an incurable cancer - an interview with Dr Francesco Crea. Epigenomics. 2019;11(13):1461–1462. 31536382 10.2217/epi-2019-0236

[15] Li JH, Liu S, Zhou H, et al. starBase v2.0: decoding miRNA-ceRNA, miRNA-ncRNA and protein-RNA interaction networks from large-scale CLIP-Seq data. Nucleic Acids Res. 2014;42(D1):D92–D97. 24297251 10.1093/nar/gkt1248 PMC3964941

[16] Gyorffy B, Lanczky A, Eklund AC, et al. An online survival analysis tool to rapidly assess the effect of 22,277 genes on breast cancer prognosis using microarray data of 1,809 patients. Breast Cancer Res Treat. 2010;123(3):725–731. 20020197 10.1007/s10549-009-0674-9

[17] Livak KJ, Schmittgen TD. Analysis of relative gene expression data using real-time quantitative PCR and the 2(T)(-Delta Delta C) method. Methods. 2001;25(4):402–408. 11846609 10.1006/meth.2001.1262

[18] Takai H, Smogorzewska A, de Lange T. DNA damage foci at dysfunctional telomeres. Curr Biol. 2003;13(17):1549–1556. 12956959 10.1016/s0960-9822(03)00542-6

[19] Yamaguchi H, Oikawa T. Membrane lipids in invadopodia and podosomes: key structures for cancer invasion and metastasis. Oncotarget. 2010;1(5):320–328. 21307399 10.18632/oncotarget.164 PMC3157727

[20] Shin SJ, Kim J, Lee S, et al. Ulipristal acetate induces cell cycle delay and remodeling of extracellular matrix. Int J Mol Med. 2018;42(4):1857–1864. 30015921 10.3892/ijmm.2018.3779 PMC6108884

[21] Kitanaka N, Nakano R, Sakai M, et al. ERK1/ATF-2 signaling axis contributes to interleukin-1beta-induced MMP-3 expression in dermal fibroblasts. PLoS One. 2019;14(9):e0222869. 31536594 10.1371/journal.pone.0222869 PMC6752866

[22] Igissinov N, Toguzbayeva A, Turdaliyeva B, et al. Breast cancer in megapolises of Kazakhstan: epidemiological assessment of incidence and mortality. Iran J Public Health. 2019;48(7):1257–1264. 31497546 PMC6708542

[23] Wein L, Loi S. Mechanisms of resistance of chemotherapy in early-stage triple negative breast cancer (TNBC). Breast. 2017;34(Suppl 1):S27–S30. 28668293 10.1016/j.breast.2017.06.023

[24] Dirix LY, Takacs I, Jerusalem G, et al. Avelumab, an anti-PD-L1 antibody, in patients with locally advanced or metastatic breast cancer: a phase 1b JAVELIN solid tumor study. Breast Cancer Res Treat. 2018;167(3):671–686. 29063313 10.1007/s10549-017-4537-5 PMC5807460

[25] Foroughi K, Amini M, Atashi A, et al. Tissue-specific down-regulation of the long non-coding RNAs PCAT18 and LINC01133 in gastric cancer development. Int J Mol Sci. 2018;19(12):3881. 10.3390/ijms19123881 PMC6321575 30518158

[26] Zhang J, Zhang H, Wang X, et al. PCAT18, as a novel differentially regulated long noncoding RNA in adult acute myeloid leukemia patients revealed by next-generation sequencing. Int J Lab Hematol. 2020;42(6):858–865. 33006448 10.1111/ijlh.13305

[27] Crea F, Watahiki A, Quagliata L, et al. Identification of a long non-coding RNA as a novel biomarker and potential therapeutic target for metastatic prostate cancer. Oncotarget. 2014;5(3):764–774. 24519926 10.18632/oncotarget.1769 PMC3996663

[28] Dou J, Tu DY, Zhao HJ, et al. LncRNA PCAT18/miR-301a/TP53INP1 axis is involved in gastric cancer cell viability, migration and invasion. J Biochem. 2020;168(5):547–555. 32687182 10.1093/jb/mvaa079

[29] Yang D, Li R, Xia J, et al. Long noncoding RNA PCAT18 upregulates SPRR3 to promote colorectal cancer progression by binding to miR-759. Cancer Manag Res. 2020;12:11445–11452. 33204157 10.2147/CMAR.S272652 PMC7667148

[30] Gozdecka M, Breitwieser W. The roles of ATF2 (activating transcription factor 2) in tumorigenesis. Biochem Soc Trans. 2012;40(1):230–234. 22260696 10.1042/BST20110630

[31] Guo HQ, Ye S, Huang GL, et al. Expression of activating transcription factor 7 is correlated with prognosis of colorectal cancer. J Cancer Res Ther. 2015;11(2):319–323. 26148593 10.4103/0973-1482.148688

[32] van Dam H, Castellazzi M. Distinct roles of Jun: fos and Jun: ATF dimers in oncogenesis. Oncogene. 2001;20(19):2453–2464. 11402340 10.1038/sj.onc.1204239

[33] Li K, Wei L, Huang Y, et al. Leptin promotes breast cancer cell migration and invasion via IL-18 expression and secretion. Int J Oncol. 2016;48(6):2479–2487. 27082857 10.3892/ijo.2016.3483

[34] Gokulnath M, Swetha R, Thejaswini G, et al. Transforming growth factor-beta1 regulation of ATF-3, c-Jun and JunB proteins for activation of matrix metalloproteinase-13 gene in human breast cancer cells. Int J Biol Macromol. 2017;94(Pt A):370–377. 27751807 10.1016/j.ijbiomac.2016.10.026

[35] Quinn JJ, Chang HY. Unique features of long non-coding RNA biogenesis and function. Nat Rev Genet. 2016;17(1):47–62. 26666209 10.1038/nrg.2015.10

[36] Tang HX, Zhu DL, Zhang GZ, et al. AFAP1-AS1 promotes proliferation of pituitary adenoma cells through miR-103a-3p to activate PI3K/AKT signaling pathway. World Neurosurg. 2019;130:E888–E898. 31299308 10.1016/j.wneu.2019.07.032

[37] Yu MJ, Xue YX, Zheng J, et al. Linc00152 promotes malignant progression of glioma stem cells by regulating miR-103a-3p/FEZF1/CDC25A pathway. Mol Cancer. 2017;16. DOI:10.1186/s12943-017-0677-9 PMC5485714 28651608

[38] He Q, Zhao L, Liu X, et al. MOV10 binding circ-DICER1 regulates the angiogenesis of glioma via miR-103a-3p/miR-382-5p mediated ZIC4 expression change. J Exp Clin Cancer Res. 2019;38(1):9. 30621721 10.1186/s13046-018-0990-1 PMC6323715

[39] Zhong ZY, Lv MX, Chen JX. Screening differential circular RNA expression profiles reveals the regulatory role of circTCF25-miR-103a-3p/miR-107-CDK6 pathway in bladder carcinoma. Sci Rep. 2016;6:30919. 10.1038/srep30919 PMC4971518 27484176

[40] Hu X, Miao J, Zhang M, et al. miRNA-103a-3p promotes human gastric cancer cell proliferation by targeting and suppressing ATF7 in vitro. Mol Cells. 2018;41(5):390–400. 29754469 10.14348/molcells.2018.2078 PMC5974616

