# Supplementary figures and images for: lncRNA prostate cancer-associated transcript 18 upregulates activating transcription factor 7 to prevent metastasis of triple-negative breast cancer via sponging miR-103a-3p
Source: Bioengineered. 2021 Dec 14;12(2):12070–86. doi: 10.1080/21655979.2021.2003928 (PMC8809992; doi:10.1080/21655979.2021.2003928)

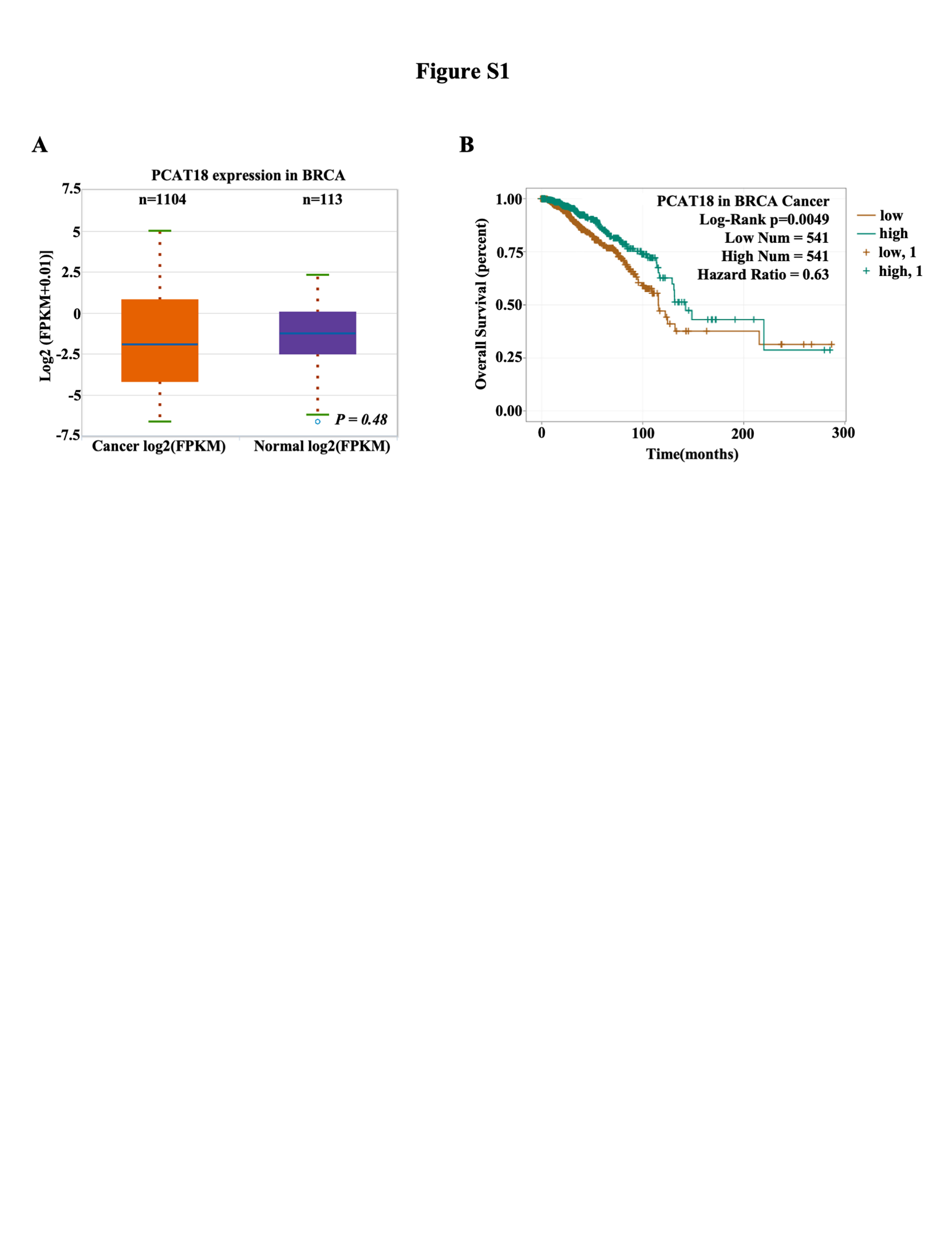

Supplement: Supplemental Material [file KBIE_A_2003928_SM2444.zip › supplementary/Figure S1.tif]

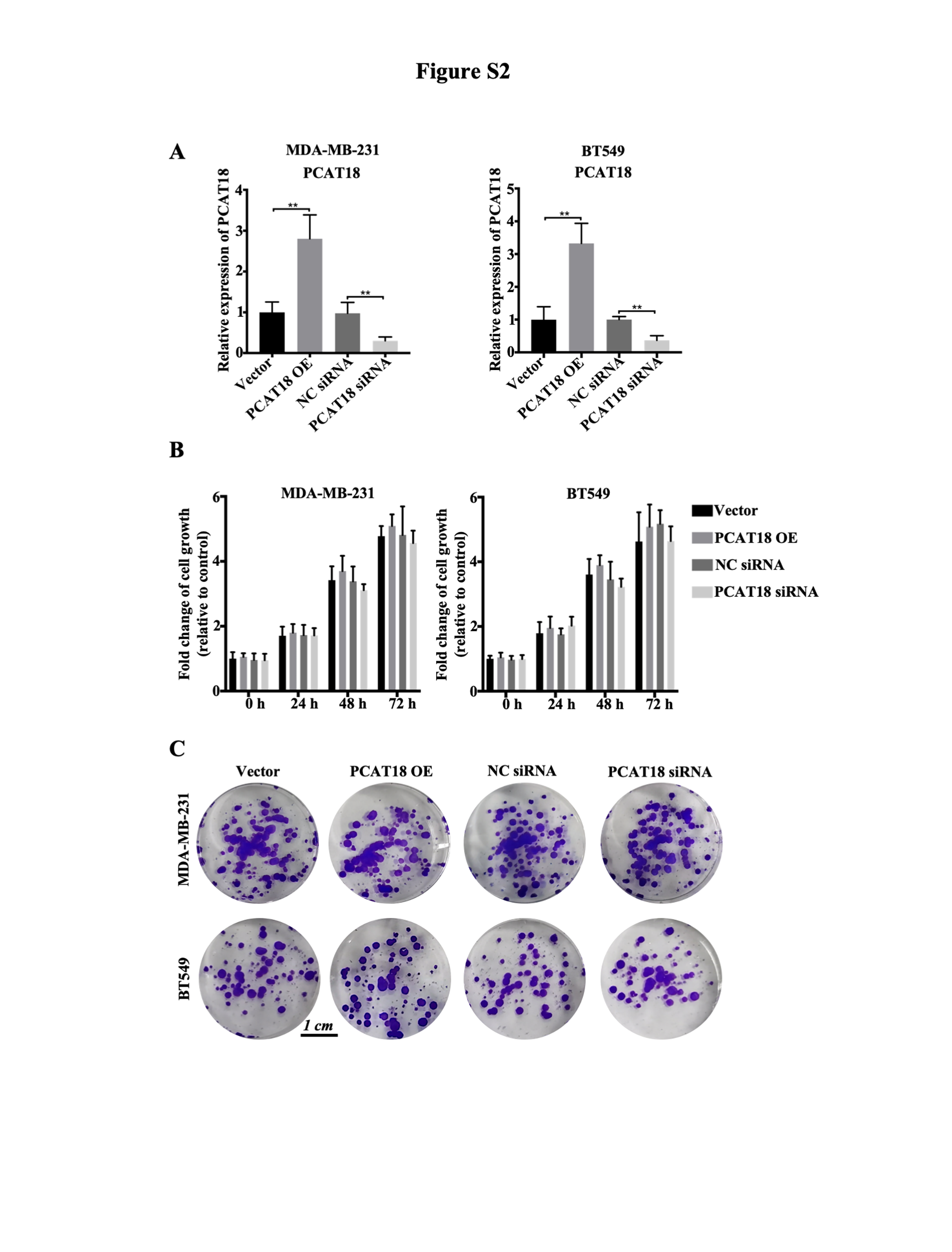

Supplement: Supplemental Material [file KBIE_A_2003928_SM2444.zip › supplementary/Figure S2.tif]

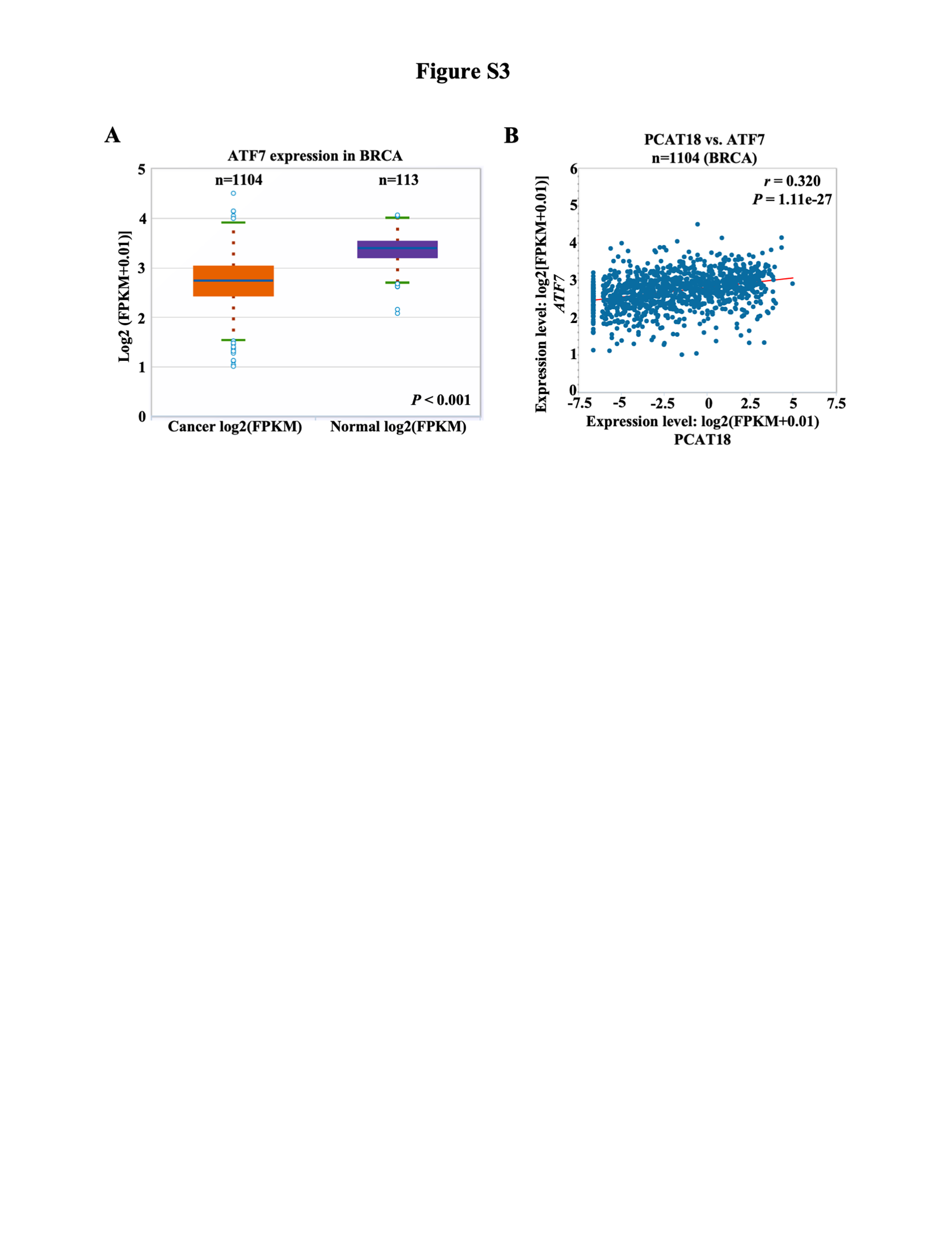

Supplement: Supplemental Material [file KBIE_A_2003928_SM2444.zip › supplementary/Figure S3.tif]

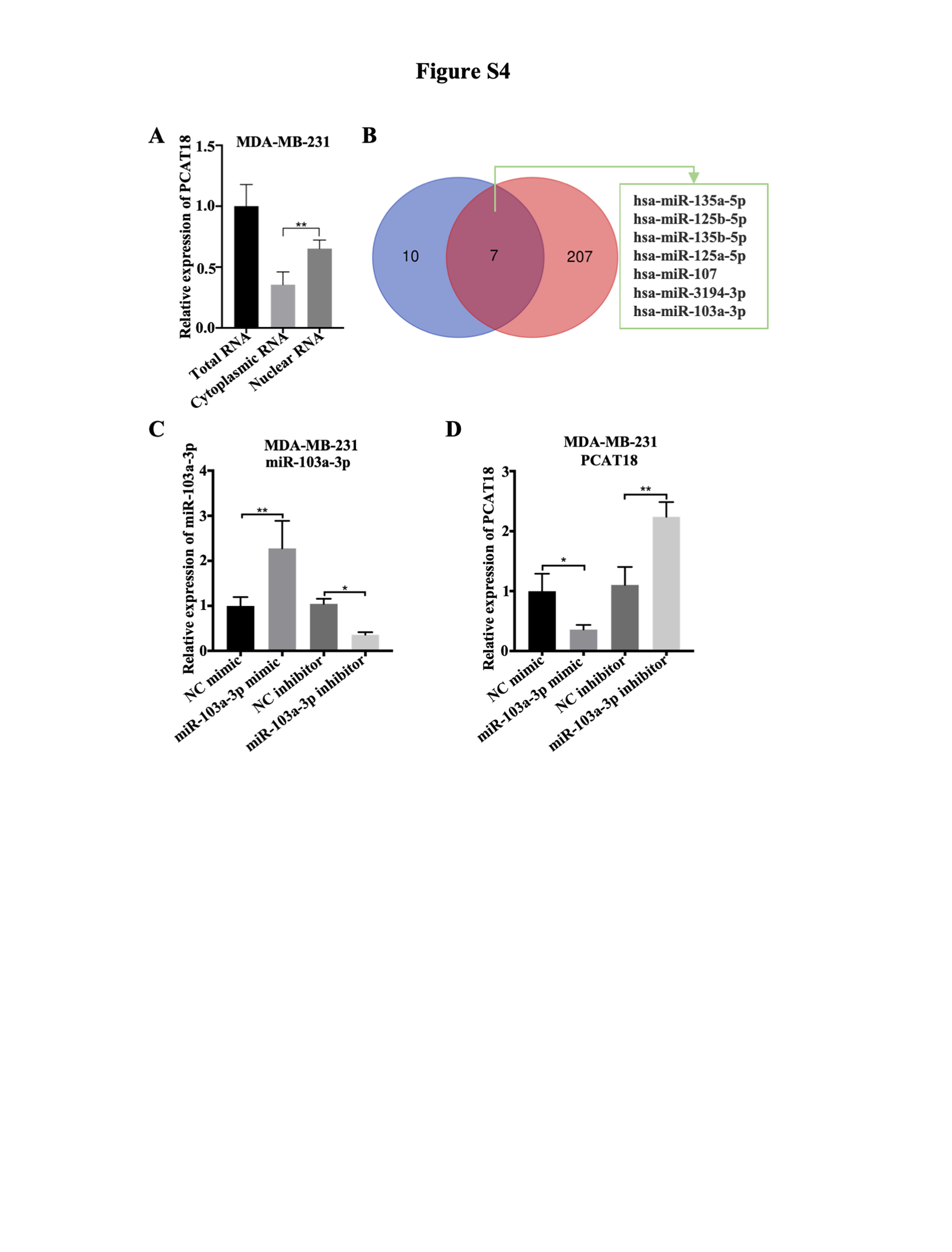

Supplement: Supplemental Material [file KBIE_A_2003928_SM2444.zip › supplementary/Figure S4.tif]
